# Correction: Gateway Vectors for Efficient Artificial Gene Assembly *In Vitro* and Expression in Yeast *Saccharomyces cerevisiae*

**DOI:** 10.1371/journal.pone.0150127

**Published:** 2016-02-19

**Authors:** The PLOS ONE Staff

There are errors in the Results, Table 2 and Table 3, which were introduced during the typesetting process. The publisher apologizes for these errors.

**Table 2:** Entry clones created in this study.

**Plasmid**	**Promoter**
pYS1	*S*. *cerevisiae CUP1*
pYS2	*S*. *pombe ADH1*
pYS3	*S*. *cerevisiae TEF*
pYS6	*TetO*_7_ -CYC1TATA
pYS7	*TetO*_2_ -CYC1TATA
**Plasmid**	**ORF**
pCG32	yEGFP
pDHM7	yEGFP-Cln2_PEST_
pCG55	yEVenus
pYS61	yEVenus-NLS*^1^
pCG98	yEVenus-Cln2_PEST_-NLS
pCG40	mCherry
pYS60	mCherry-NLS
pYS19	tTA
pYS20	rtTA
pYS58	AIDtTA
pYS57	AIDrtTA
pCG72	OsTIR1-9Myc

Promoter Entry clones were created with pDONR221P5-P2 and ORF Entry clones with pDONR221P1-P5r.

*^1^ Significant cytoplasmic fluorescence was observed when overexpressed, for example, by *TEF* promoter.

**Table 3:** Expression plasmid vectors constructed by Gateway recombination method in this study.

Plasmid	Promoter	ORF	Marker	Destination vector
pCG52	*S*. *cerevisiaeTEF*	mCherry	*LEU2*	pDEST415TEFt7
pCG109	*S*. *pombe ADH1*	mCherry-NLS	*TRP1*	pDEST414TEFt7
pCG57	*S*. *cerevisiae TEF*	yEVenus	*LEU2*	pDEST415TEFt7
pRN1	*S*. *pombe ADH1*	yEVenus-NLS	*LEU2*	pDEST415TEFt7
pDHM57	*S*. *pombe ADH1*	yEVenus-Cln2_PEST_-NLS	*LEU2*	pDEST415TEFt7
pCM25	*S*. *cerevisiae CUP1*	yEVenus-Cln2_PEST_-NLS	*LEU2*	pDEST415TEFt7
pCG87	*TetO*_7_-CYC1TATA	mCherry-NLS	*TRP1*	pDEST414TEFt7
pCG103	*TetO*_7_-CYC1TATA	yEVenus-Cln2_PEST_-NLS	*TRP1*	pDEST414TEFt7
pCM20	*TetO*_7_-CYC1TATA	yEVenus-Cln2_PEST_-NLS	*LEU2*	pDEST415TEFt7
pCG84	*S*. *pombe ADH1*	tTA	*HIS3*	pDEST413TEFt7
pCG85	*S*. *pombe ADH1*	rtTA	*HIS3*	pDEST413TEFt7
pDHM19	*S*. *pombe ADH1*	AIDtTA	*HIS3*	pDEST413TEFt7
pDHM20	*S*. *pombe ADH1*	AIDrtTA	*HIS3*	pDEST413TEFt7
pCG112*^1^	*S*. *cerevisiae TEF*	tTA	*HIS3*	pDEST413TEFt7
pCG113	*S*. *cerevisiae TEF*	rtTA	*HIS3*	pDEST413TEFt7
pCG106*^1^	*S*. *cerevisiae TEF*	AIDtTA	*HIS3*	pDEST413TEFt7
pCG107	*S*. *cerevisiae TEF*	AIDrtTA	*HIS3*	pDEST413TEFt7
pMM6*^2^	*S*. *cerevisiaeTEF*	AIDrtTA	*URA3*, *MET15*	pDEST375
pCG81	*S*. *pombe ADH1*	OsTIR1-9Myc	*URA3*	pDEST416TEFt7

*^1^ Yeast cells with these expression vectors showed poor growth with an unknown reason.

*^2^ Integration vector.

There is an error in the penultimate sentence of the third paragraph of the “Construction of Yeast Gateway Vectors for One-step Gene Assembly” subsection of the Results. The correct sentence is: We compared the fluorescence produced by yEVenus-Cln2_PEST_-NLS and yEVenus-NLS when expressed by the constitutive promoter ADH1 (Fig 4B, C).

There is an error in Table 2, “Entry clones created in this study.” Please see the corrected Table 2 here.

There is an error in Table 3, “Expression plasmid vectors constructed by Gateway recombination method in this study.” Please see the corrected Table 3 here.
